# Potential Neuroprotective Effects of *Alpinia officinarum Hance* (Galangal): A Review

**DOI:** 10.3390/nu16193378

**Published:** 2024-10-04

**Authors:** Izzat Zulhilmi Abd Rahman, Siti Hajar Adam, Adila A. Hamid, Mohd Helmy Mokhtar, Ruslinda Mustafar, Mohd Izhar Ariff Mohd Kashim, Ami Febriza, Nur Izzati Mansor

**Affiliations:** 1 Department of Physiology, Faculty of Medicine, Universiti Kebangsaan Malaysia, Kuala Lumpur 56000, Malaysia; izzat_zulhilmi@ukm.edu.my (I.Z.A.R.); adilahamid@ppukm.ukm.edu.my (A.A.H.); helmy@ukm.edu.my (M.H.M.); 2 Preclinical Department, Faculty of Medicine & Defence Health, Universiti Pertahanan Nasional Malaysia, Kuala Lumpur 57000, Malaysia; siti.hajar@upnm.edu.my; 3 Department of Medicine, Faculty of Medicine, Universiti Kebangsaan Malaysia, Kuala Lumpur 56000, Malaysia; ruslinda.mustafar@ppukm.ukm.edu.my; 4 Centre of Shariah, Faculty of Islamic Studies, Universiti Kebangsaan Malaysia, Bangi 43600, Malaysia; izhar@ukm.edu.my; 5 Institute of Islam Hadhari, Universiti Kebangsaan Malaysia, Bangi 43600, Malaysia; 6 Faculty of Medicine and Health Sciences, Universitas Muhammadiyah Makassar, Makassar 90221, South Sulawesi, Indonesia; amifebriza@med.unismuh.ac.id; 7 Department of Nursing, Faculty of Medicine, Universiti Kebangsaan Malaysia, Kuala Lumpur 56000, Malaysia

**Keywords:** *Alpinia officinarum Hance*, galangal, central nervous system, neurological disorders, neuroprotective, therapeutic potentials

## Abstract

**Background/Objectives:** This review aims to provide a detailed understanding of the current evidence on *Alpinia officinarum Hance* (*A. officinarum*) and its potential therapeutic role in central nervous system (CNS) disorders. CNS disorders encompass a wide range of disorders affecting the brain and spinal cord, leading to various neurological, cognitive and psychiatric impairments. In recent years, natural products have emerged as potential neuroprotective agents for the treatment of CNS disorders due to their outstanding bioactivity and favourable safety profile. One such plant is *A. officinarum*, also known as lesser galangal, a perennial herb from the Zingiberaceae family. Its phytochemical compounds such as flavonoids and phenols have been documented to have a powerful antioxidants effect, capable of scavenging free radicals and preventing oxidative damage. **Methods:** In this review, we critically evaluate the in vitro and in vivo studies and examine the mechanisms by which *A. officinarum* exerts its neuroprotective effect. **Results:** Several studies have confirmed that *A. officinarum* exerts its neuroprotective effects by reducing oxidative stress and cell apoptosis, promoting neurite outgrowth, and modulating neurotransmitter levels and signalling pathways. **Conclusions:** Although previous studies have shown promising results in various models of neurological disorders, the underlying mechanisms of *A. officinarum* in Alzheimer’s (AD) and Parkinson’s disease (PD) are still poorly understood. Further studies on brain tissue and cognitive and motor functions in animal models of AD and PD are needed to validate the results observed in in vitro studies. In addition, further clinical studies are needed to confirm the safety and efficacy of *A. officinarum* in CNS disorders.

## 1. Introduction

*Alpinia officinarum Hance* (*A. officinarum*), also known as lesser galangal, is a perennial herb that belongs to the Zingiberaceae family [1]. The Zingiberaceae family is native to southern China and comprises 53 genera and 1300 species that are widely distributed in the tropics of Africa, the Americas and Asia, especially in India, Indonesia, Malaysia, Egypt, Sri Lanka, Vietnam and Thailand due to their favourable agro-ecological conditions [2,3,4]. This plant naturally thrives in moist, tropical environments with extensive sunlight exposure but can also grow in shrubs, forests and open areas [5].

**Figure 1:**
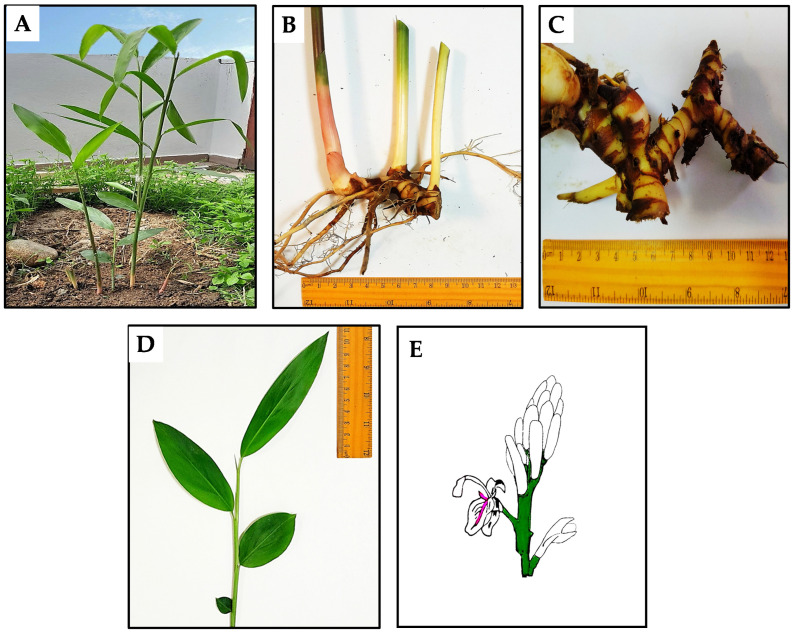
The various parts of *A. officinarum*. (**A**) the whole plant; (**B**) roots and rhizome; (**C**) rhizome; (**D**) leaves; and (**E**) illustration of the flower of *A. officinarum*.

*A. officinarum* is a herbaceous plant that can grow up to 150 cm tall, characterized by hard, elongated, cylindrical, subterranean rhizomes and small adventitious roots. The rhizomes display a reddish-brown exterior, while the interior varies from brown to orange. These rhizomes are typically between 2.5 and 10 cm long. The plant has an erect pseudostem, sheathed by linear-lanceolate leaves that are between 20 and 30 cm long and 1.2 and 2.5 cm wide. They are glabrous, distichous and pointed at the tips. The compound inflorescences measure 3 to 4 cm in length and emit a pleasant scent. The flowers have green corolla bases and white buds, arranged in racemes. The fruit is an ellipsoidal, capsule-shaped structure with a diameter of approximately 1.5 cm, showing a color gradient from orange to red. The flowering period is from April to September, and the fruiting period takes place from May to November [5,6,7,8]. The various parts of the plant are shown in Figure 1.

The rhizome of *A. officinarum* is the part that has been most extensively studied for its medicinal properties. However, the specific composition of the leaves and roots may vary, and further research is required to fully understand its nutritional properties. There is also limited information on the composition of the flowers of *A. officinarum*.

The global demand for *A. officinarum* has steadily increased due to its increasing use in various sectors. *A. officinarum* is valued for its anti-inflammatory, antioxidant and neuroprotective properties, which favours its use in herbal preparations [9]. For over a millennium, *A. officinarum* has been traditionally used in various cultures such as Chinese, Ayurvedic, Thai and Greek medicine to improve blood circulation, reduce swelling, relieve stomach pain, diarrhoea, nausea, diabetes, headaches, kidney problems, ulcers, joint pain and respiratory problems [10]. Although *A. officinarum* is known for its medicinal properties, it is also used in culinary practices as a flavor enhancer and preservative. Its unique sweet and spicy aroma makes it a popular pickling spice in Asian cuisine, improving both flavor and shelf life [11]. In addition, the modern cosmetics industry has increasingly turned to natural ingredients for their potential skin-protecting effects [12]. *A. officinarum* extracts have shown considerable sun protection factor (SPF) and free radical scavenging activities, which increases their effectiveness as natural sunscreens [13].

Economically, the World Health Organization’s endorsement of traditional medicine, particularly in developing nations, has contributed to the exponential expansion of the herbal medicine sector worldwide [14]. The global market for medicinal plants is estimated at over USD 60 billion and is expected to grow to USD 5 trillion by 2050 [15]. The Asia-Pacific region, particularly China and India, plays a pivotal role in the global herbal market. China alone exports 120,000 tonnes of herbal remedies every year [16].

Recent studies have demonstrated its diverse pharmacological properties and supported its traditional uses. *A. officinarum* contains potent antioxidants such as flavonoids and phenolics which help reduce oxidative stress by scavenging free radicals [17]. Its anti-inflammatory and analgesic effects are widely reported and possibly operate by inhibiting pro-inflammatory cascades such as nuclear factor kappa B/tumour necrosis factor-α (NF-κB/TNF-α), cyclooxygenase-2 (COX-2), p38 mitogen-activated protein kinases and NOD-like receptor protein 3 (NLRP3) [18,19]. Remarkably, *A. officinarum* also demonstrates significant antidiabetic activity, primarily by interfering with the α-glucosidase enzyme to reduce the conversion of carbohydrates to glucose [20]. Furthermore, Fu et al. showed that *A. officinarum* possesses antibacterial properties by altering the hydrophobicity of the bacterial cell surface and membrane permeability, thereby enhancing its bactericidal activity [21]. Preliminary studies suggest that ethyl-p-methoxycinnamate in galangal exhibits anti-proliferative and cytotoxic effects on human cervical and breast cancer cells, indicating its potential anticancer properties [22].

Central nervous system (CNS) diseases encompass a diverse spectrum of conditions that affect the brain and spinal cord, leading to various neurological and cognitive impairments [23]. These diseases can be broadly classified into several categories: infectious (e.g., meningitis, encephalitis and brain abscess), inflammatory (e.g., multiple sclerosis, neuromyelitis optical spectrum and transverse myelitis) [24], neurodegenerative (e.g., Alzheimer’s disease, Parkinson’s disease, Huntington’s disease and amyotrophic lateral sclerosis) [25], developmental (e.g., epilepsy, cerebral palsy, autism and schizophrenia) [26] and neoplastic disorders (e.g., glioma, astrocytoma and neuroblastoma) [27].

CNS diseases remain a major health challenge, particularly in low- and middle-income countries, as regional access to screening, diagnosis and treatment is inadequate [28]. Over the last 30 years, mortality rates have increased by 39% and disability-adjusted life-years (DALYs) by 15%, although the prevalence of neurological diseases has decreased, so urgent measures and action plans are needed [29]. Neurological impairments adversely affect patients’ well-being, reducing their job performance and productivity. This leads to significant financial losses, which can be considered as a reduction in human capital [30]. Consequently, the economic impact of absenteeism from work due to chronic disease-related functional limitations in the working-age population is estimated to be around 4.95 billion annually [31].

Surprisingly, modern treatment of CNS diseases still faces major challenges due to the complexity of the brain and its protective mechanisms [32]. Neurodegenerative diseases are particularly difficult to treat due to the lack of disease-modifying therapies, and the blood–brain barrier (BBB) limits effective drug delivery to the brain [33]. CNS infections also require rapid diagnosis and treatment. However, conventional diagnostic methods are often slow and inaccurate, leading to treatment delays and an increased risk of complications [34]. Psychiatric disorders are even more complex due to the limited understanding of their pathophysiology. There are currently no objective diagnostic biomarkers available, complicating the treatment options. Approximately 93% of drug candidates fail, partly due to their non-synergistic interaction with the brain’s neural system, resulting in limited effectiveness and adverse effects [35].

To date, the number of comprehensive reviews dealing with different aspects of *A. officinarum* has increased significantly. These reviews have investigated numerous topics, including the phytochemical and safety profiles of *A. officinarum*, as well as its pharmacological effects and therapeutic applications [8,36,37,38,39]. Several reviews have established a correlation between extraction/isolation techniques and phytochemical composition and biological activity. For example, Basri et al. [8] and Ahmed et al. [38] reviewed the findings from previous studies on the phytochemical composition and biological activities of various solvent extracts, fractions and isolated compounds from different plant parts of *A. officinarum*, such as the rhizomes, leaf, aerial part and roots. The chemical composition and biological activities of the various *Alpinia* species were also analysed and compared. In the review by Zou et al., the photochemistry and pharmacology of two plant species, namely *Alpinia galanga* (L.) Willd and *A. officinarum* were discussed [6]. Meanwhile, another review highlighted the chemical composition and biological activities of essential oils from the *Alpinia* genus [40]. In another review, reports on the safety and toxicity profile of *A. officinarum* were lacking. However, the oil of *A. officinarum* was reported to be acutely toxic and cause mild skin irritation in rabbits for up to 24 h. The authors also summarised the safety and toxicity profile of Ankaferd Blood Stopper^®®^, a combination of herbal extracts containing *A. officinarum*, in randomised clinical trials in healthy volunteers and found it to be safe [9]. Lei et al. analysed the traditional uses of *A. officinarum* and identified 337 compounds reported in previous studies. In that review, the crude extract of *A. officinarum* and its compounds were reported to exert a wide range of biological activities, including improved gastrointestinal function, anti-inflammatory, analgesic and antitumour effects, antibacterial properties and memory enhancement [39].

While the phytochemical constituents, biological activities and safety profile of *A. officinarum* have been extensively examined in previous publications, the potential effects *A. officinarum* on CNS diseases, such as Parkinson’s disease, depression, epilepsy and cerebral ischaemia, have not yet been adequately addressed. To the best of our knowledge, only the review by Mukherjee et al. examines the properties and use of *A. officinarum* as a traditional medicine for various diseases, with a focus on its function in Alzheimer’s disease [41]. In recent years, there is a growing interest in natural products as potential neuroprotective agents, attributed to their diverse bioactive compounds and favourable safety profiles [42]. Therefore, a comprehensive review of the neuroprotective effects of *A. officinarum* is warranted. Furthermore, in light of new research findings, there is an urgent need for an updated synthesis of the evidence on the benefits of *A. officinarum* in relation to CNS diseases. The aim of this review is to provide a detailed understanding of current evidence, highlight existing research gaps and emphasise the neuroprotective properties of *A. officinarum* and its mechanism of action in CNS disorders which have not been fully addressed in previous work. This could pave the way for further preclinical and clinical studies and ultimately contribute to the development of new, effective treatments for these diseases.

## 2. Methodology

**Figure 2:**
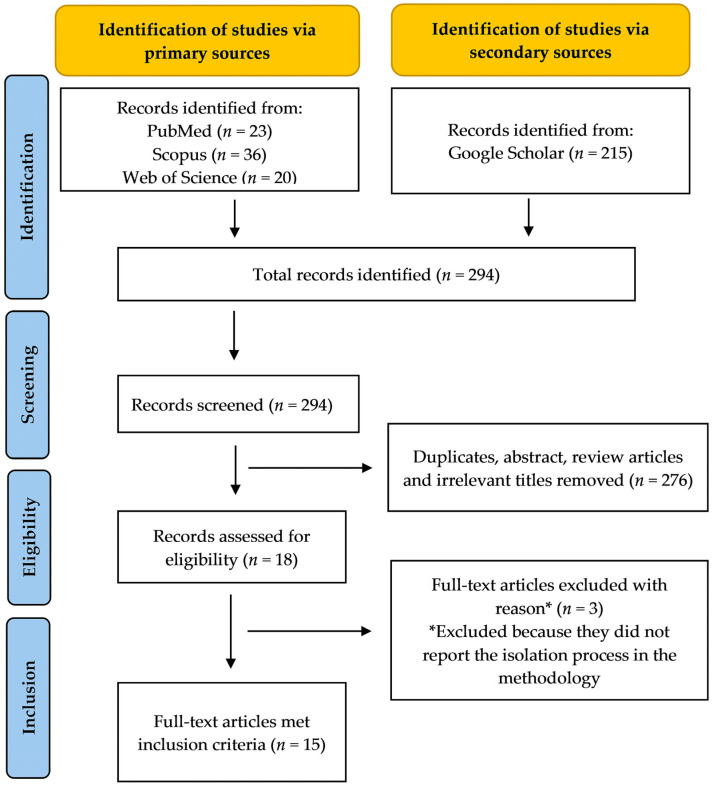
Flowchart of the article selection process.

The relevant studies were obtained from four major databases: Google Scholar, Scopus, PubMed and Web of Science (WoS). The following keywords were used: *Alpinia officinarum* AND (central nervous system OR brain OR neuron OR neurological disorders OR Parkinson’s disease OR Alzheimer’s disease OR depression OR epilepsy OR cerebral ischaemia). All clinical, in vivo and in vitro studies investigating the effects of *Alpinia officinarum* (*A. officinarum*) on CNS-related models were considered, regardless of the route of administration, type of preparation (plant extract or bioactive ingredient), dose or duration of intervention. For studies with bioactive constituents, only studies that described the isolation process in the methodology were considered. Studies that used a combined preparation of *A. officinarum* with other plants were excluded. Figure 2 summarises the flowchart of the article selection process.

Throughout the article selection process, a total of 294 records were identified from primary sources (PubMed, Scopus and Web of Science) and secondary sources (Google Scholar). After screening the titles and abstracts, 276 records were excluded due to duplication, irrelevance or being review articles. The remaining 18 full-text articles were assessed for eligibility, and 3 were excluded for not reporting the isolation process in their methodology. Ultimately, 15 studies satisfied the inclusion criteria and were included in this review. The following information was extracted from the studies: the type of sample preparation, the experimental models for neurological disorders, the parameters investigated and the analysis technique, as well as the results and conclusions of the studies.

## 3. Phytochemistry and Constituents of *Alpinia officinarum Hance* (Galangal)

Extensive research on *A. officinarum* has revealed the presence of numerous phytochemical compounds, mainly isolated from the rhizomes. These compounds include phenylpropanoids, flavonoids, diarylheptanoids, glycosides, sesquiterpenes and diterpenes. Phenolic compounds, especially phenylpropanoids, are abundant in *A. officinarum*. Ly et al. isolated seven phenylpropanoids from *A. officinarum* that exhibited antioxidant activity against the autoxidation of methyl linoleate in the bulk phase [43]. Galangal is also rich in diarylheptanoids and flavonoids, mainly galangin, kaempferide and kaempferol [44]. These groups of compounds have been demonstrated to exhibit a range of bioactivities including antioxidant, α-glucosidase, butyrylcholinesterase and acetylcholinesterase inhibition in vitro, suggesting its therapeutic potential [45].

**Figure 3:**
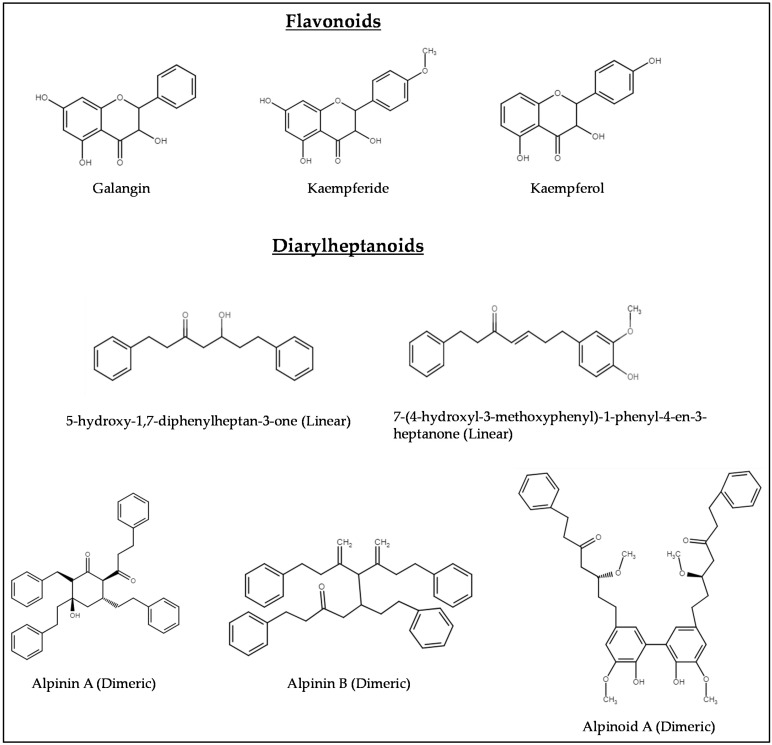
Chemical structures of flavonoids and diarylheptanoids from *A. officinarum* which are commonly reported for their biological activities.

Diarylheptanoids, another group of frequently isolated phytochemicals from the rhizome, are the most characteristic and important active constituents of *A. officinarum*. These compounds consist of two aromatic rings (aryl groups) linked by a heptyl chain with different substituents. Linear diarylheptanoids have a straight, unbranched seven-carbon chain connecting the two aromatic rings; cyclic diarylheptanoids have a seven-carbon chain that forms a ring structure or contains additional ring formations; dimeric diarylheptanoids are involved in the dimerisation of diarylheptanoid units, resulting in a compound with two linked diarylheptanoid structures; and the novel diarylheptanoids are the new or lesser-known diarylheptanoids that may have unique structures or properties that have not been previously documented. In this review, the potential neurological effects identified are primarily attributed to the diarylheptanoids and flavonoids. A summary of the phytochemical compounds isolated from *A. officinarum* can be found in Table 1. Figure 3 presents the chemical structures of flavonoids and diarylheptanoids from *A. officinarum* which are commonly reported for their biological activities.

Several studies explored phytochemical compounds and biological activities in the different parts of *A. officinarum*. Interestingly, the phytochemical composition and biological activities of *A. officinarum* are not always the same and could be influenced by the different parts of the plant and the extraction or isolation techniques. Previous studies reported that the rhizomes have a higher concentration of compounds than the aerial parts of *A. officinarum*. Solvent extracts have shown significant biological activity, including antioxidant, anti-inflammatory, anti-cancer and antibacterial properties, due to the presence of diarylheptanoids and flavonoids in *A. officinarum* [6,38]. Table 2 summarises the reported phytochemical compounds isolated from different parts of the *A. officinarum* plant and their biological activities.

## 4. Effects of *Alpinia officinarum Hance* (Galangal) on Central Nervous System (CNS)

The extract of *A. officinarum* and its bioactive constituents have been extensively studied in in vitro and in vivo models for their pharmacological activities on the CNS, such as neuritogenic, neurogenic, anticholinesterase, antidepressant, anticonvulsant, analgesic and memory enhancing/nootropic activities. These effects are attributed to the presence of diarylheptanoids and flavonoids in *A. officinarum*, which are often associated with antioxidant and anti-inflammatory activities.

### Effects of A. officinarum on Enhancement and Regeneration of Neuronal Cells

In recent years, the stimulation of neuronal differentiation and adult neurogenesis in the brain has been proposed as a promising approach for the treatment of neurological disorders such as neurodegenerative diseases, traumatic brain injury and mental illness. Previous studies have investigated the neuritogenic and neurogenic effects of diarylheptanoids from *A. officinarum* on neuronal cell lines. Interestingly, these compounds were initially tested for their anticancer potential against neuroblastoma cells [68,69]. However, the findings led the researchers to a different potential of diarylheptanoids, as they also promote neuronal differentiation in neuroblastoma cells. Tabata et al. [68], for example, investigated the antitumour effect of two diarylheptanoids, 7-(4-hydroxy-3-methoxyphenyl)-1-phenyl-4E-hepten-3-one (Cpd 1) and (5R)-5-methoxy-7-(4-hydroxy-3-methoxyphenyl)-1-phenyl-3-heptanone (Cpd 2), derived from *A. officinarum* on neuroblastoma cells (IMR-32, SK-N-SH and NB-39 cells) in vitro. The authors found that both compounds exhibited cytotoxic activity against neuroblastoma cells. At the same time, Cpd 1 induced the differentiation of neuroblastoma cells, as evidenced by increased neurite length and branching in undifferentiated NB-39 cells [68]. These findings have led to further investigations into the neuritogenic potential of diarylheptanoids from *A. officinarum*. A study by Tang et al. demonstrated the ability of Cpd 1 and Cpd 2 from *A. officinarum* to induce neuronal differentiation in mouse neuroblastoma cells (Neuro-2a) and promote axon–dendrite polarisation in cultured rat hippocampal neurons, which was characterised by enhanced neurite outgrowth, an increased percentage of axon-bearing cells and increased expression of neurofilament M protein, a marker for mature neurons [55]. In parallel, the authors also discovered that a 14-day treatment with Cpd 1 (28 mg/kg BW) in healthy mice increased the differentiation and maturation of newborn progenitor cells in the adult dentate gyrus. The study also showed that the neuritogenic and neurogenic effects of Cpd 1 in promoting neuronal differentiation and neurogenesis were regulated by extracellular signal-regulated kinase (ERK) and phosphatidylinositol 3-kinase (PI3K)/AKT signalling pathways [55]. Taken together, these results demonstrate the neuroenhancing and neuroregenerative effects of *A. officinarum* and its potential as an alternative strategy for the treatment of neurological disorders. Table 3 summarises the effects of *A. officinarum* on neuronal cells in in vitro and in vivo studies.

## 5. Effects of *A. officinarum* on Neurological Disorders

Numerous in vitro and in vivo studies have shown that preparations of *A. officinarum* and its bioactive compounds have a protective effect on neurological disorders, particularly Alzheimer’s disease (AD) and Parkinson’s disease (PD), depression, epilepsy and cerebral ischaemia-reperfusion injury.

### 5.1. Effect of A. officinarum on Alzheimer’s Disease

Alzheimer’s disease (AD) is the most common form of neurodegenerative dementia in older people [70]. Clinically, AD is characterised by a gradual decline in memory and cognitive abilities, altered behaviours such as paranoia, hallucinations, and a progressive deterioration in language function [71,72]. The abnormal aggregation of amyloid-beta (Aβ) peptides in the brain has been identified as a key factor in the pathogenesis of AD. Studies have shown that high concentrations of soluble Aβ_1-42_ oligomers in the brain can cause oxidative stress, inflammation and mitochondrial dysfunction, resulting in synapse loss and neuronal death [73,74]. Current neuroprotective strategies to protect and repair neurons from progressive cell dysfunction and death include the promotion of neuronal growth and function and the inhibition of neurotoxic events. In recent years, *A. officinarum* has been found to possess significant neuroprotective properties in the prevention of Alzheimer’s pathogenesis. In this review, we found five in vitro studies in which the neuroprotective effect of *A. officinarum* against AD was investigated.

Several studies have demonstrated the neuroprotective effect of *A. officinarum* against Aβ-induced neurotoxicity through the suppression of Aβ-induced events, including ROS formation, apoptosis and inhibition of the PI3K/mammalian target of rapamycin (mTOR) signalling pathway [75,76]. Huang et al. [75] showed that treatment with 7-(4-hydroxyphenyl)-1-phenyl-4E-hepten-3-one isolated from *A. officinarum* attenuated Aβ42-induced cell damage in cultured hippocampal neurons by suppressing apoptosis and oxidative stress. This anti-apoptotic activity was dependent on the activation of the PI3K/mTOR signalling pathway [75]. Similar results were reported by Xiao et al., who observed the neuroprotective effect of 7-(4-hydroxy-3-methoxyphenyl)-1-phenyl-4E-hepten-3-one from *A. officinarum* against Aβ42-induced in PC12 cells [76].

On the other hand, the inhibitory properties of anticholinesterase (AChE) of *A. officinarum* have also been demonstrated. An abnormally elevated AChE level is another pathological feature of AD that is associated with impaired cholinergic transmission and cognitive decline. Several attempts have been made to restore impaired cholinergic neurotransmission in the brain, including the use of acetylcholinesterase inhibitors [77]. Interestingly, Kose et al. discovered that all water (WEG), ethanol (EEG) and water/ethanol (WEEG) extracts of *A. officinarum* exhibited stronger AChE inhibitory activity than tacrine, a standard AChE inhibitor in the in vitro assay [78]. Lee et al. [56] investigated the potential anti-cholinesterase effects of six diarylheptanoids and two flavonoids isolated from *A. officinarum* in vitro. In that study, (−)-alpininoid B, a diarylheptane–monoterpene conjugate, showed strong inhibitory activity against AChE. For butyrylcholinesterase (BChE) activities, (−)-alpininoid B showed moderate inhibition, while the other compounds showed weak or no inhibitory activity. The authors also found that this compound competitively binds to the active site of AChE and thus suppresses the catalytic activity of AChE. The stable binding energies and interactions between this compound and AChE were further confirmed by molecular dynamics studies [56].

In addition, the antioxidant capacity of *A. officinarum* has been investigated as a potential agent for preventing or halting the onset of AD. Mu et al. [79] found that several diarylheptanoid compounds from *A. officinarum* (compounds **7**, **10**, **12**, **20**, **22**, **25**, **28**, **33**, **35**, **37** and **42**) at a concentration of 5, 10 or 20 μM restored the cell viability of hydrogen peroxide (H_2_O_2_)-induced SH-SY5Y cells. Furthermore, compounds **10**, **22**, **25** and **33** were found to protect SH-SY5Y cells from H_2_O_2_-induced oxidative stress by reducing the production of malondialdehyde (MDA) and nitric oxide (NO) and lowering the concentration of reactive oxygen species (ROS) [79]. These results suggest that *A. officinarum* may be effective in the treatment of AD due to its remarkable ability to reduce neurotoxicity induced by Aβ and oxidative stress and inhibit AChE activity. However, further in vivo and clinical studies are needed to confirm its safety and therapeutic efficacy. Table 4 summarises the effects of *A. officinarum* on Alzheimer’s disease in in vitro studies.

### 5.2. Effect of A. officinarum on Parkinson’s Disease

Parkinson’s disease (PD) is a motor disorder and has been shown to be the second most common form of ND in older people. The main clinical manifestations of PD include resting tremor, bradykinesia and rigidity [80]. The pathological hallmark of PD is the loss of dopaminergic (DA) neurons, i.e., dopamine-producing neurons in the substantia nigra of the midbrain, which are responsible for controlling numerous brain functions, including voluntary movements, cognitive processes and a wide range of behavioural processes such as mood, addiction and stress [81,82,83]. Many herbal remedies have shown promise in protecting and attenuating neuronal cell death in PD.

In recent years, *A. officinarum* has been investigated for its potential in the treatment of PD. We found two in vitro studies investigating its neuroprotective effect (Table 5). A study by Liu et al. [84] demonstrated the neuroprotective effect of diarylheptanoid monoterpene adduct enantiomers from *A. officinarum*, such as (±)-alpininoid A, against 1-methyl-4-phenylpyridinium (MPP+)-induced cortical neurons. MPP+ is a neurotoxin commonly used to develop in vitro models of PD. The study found that treatment with (+)-alpininoids A at a concentration of 16 μM significantly restored the cell viability of cortical neurons [84].

Another hallmark of PD is the aggregation of alpha (α)-synuclein, a presynaptic neuronal protein that regulates the release of neurotransmitters from synaptic vesicles in the brain [85]. The α-synuclein is a major component of Lewi bodies, and an excess of this protein can have deleterious effects on neurons, such as neuronal dysfunction and cell death [86,87]. α-synuclein aggregation is a key factor in the development of PD and is responsible for dopaminergic neurotoxicity. *A. officinarum* has been shown to inhibit α-synuclein aggregation in an in vitro study. Fu et al. [88] investigated the inhibitory effect of new isolated diarylheptanoids from *A. officinarum*, namely alpinin A and alpinin B, against α-synuclein aggregation in vitro. The study showed that alpinin A and alpinin B at a concentration of 10 µM inhibited α-synuclein aggregation by 66% and 67%, respectively. It has been hypothesised that the presence of a 3, 4-dihydroxy group on the benzene ring is crucial for this inhibitory activity [88]. Although these studies have shown the neuroprotective effects of *A. officinarum* in PD, the mechanisms underlying these effects have not yet been investigated.

**Table 5:** Summary of the effects of *A. officinarum* on Parkinson’s disease in in vitro studies.

Study Design	Plant Extract/Bioactive Compound	Treatment Dosage	Duration of Study	Findings	References
In vitro studies					
Primary cortical neuron induced with MPP+ (1-methyl-4-phenylpyridinium) for 20 h.	Diarylheptanoids: Alpininoids A [(+)-1]	4, 8, 16 and 32 μM	12 h	↑ cell viability.	[84]
α-synuclein aggregation assay.	Diarylheptanoids: Alpinin A and Alpinin B	10 µM	-	Inhibits α-synuclein aggregation.	[88]

Upward arrow (↑) indicate increased.

### 5.3. Effect of A. officinarum on Ischaemia-Reperfusion Injury

Ischaemia-reperfusion injury is characterised by an acute interruption of the blood supply to the tissue, resulting in tissue damage due to glucose and oxygen deprivation, followed by restoration of perfusion leading to further tissue damage. CNS ischaemia-reperfusion injury is a common feature of acute ischaemic stroke [89,90]. Ischaemia-reperfusion injury in the CNS is associated with several pathological processes, including glutamate excitotoxicity, neuronal apoptosis, oxidative stress, inflammation, BBB breakdown, cerebral oedema and angiogenesis, ultimately leading to neurological impairment [91,92,93,94,95]. Ample evidence suggests that autophagy is activated in response to ischaemia and has been studied as a therapeutic target. Autophagy is essential for the maintenance of cellular homeostasis through the degradation and recycling of proteins and damaged organelles via the autophagy–lysosomal pathway [96]. It is still controversial whether the activation of autophagy triggered by ischaemia-reperfusion has neuroprotective or deleterious effects [97,98]. However, previous studies suggest that inhibition of excessive autophagy during ischaemia-reperfusion in rats can protect neurons from apoptosis [99,100]. In addition, the AKT/mTOR signalling pathway has been identified as a potential target for the treatment of ischaemic stroke. Activation of the AKT/mTOR signalling pathway has been reported to suppress autophagy and protect neurons from apoptosis [101,102].

The beneficial effects of *A. officinarum* on the protection of neurons from ischaemia-reperfusion injury were confirmed in two in vitro studies (Table 6) using an oxygen-glucose deprivation and re-oxygenation (OGD/R) model in cultured neurons. The OGD/R model in cultured neurons is the most commonly used in vitro model to mimic ischaemia-reperfusion injury [103]. In a study conducted by Shi et al. [104], pretreatment with hydroxy-3-methoxyphenyl)-1-phenyl-4E-hepten-3-one (AO-2; 0.25, 0.5 or 1 µM) from *A. officinarum* for 2 h significantly protected cultured cortical neurons from OGD/R-induced damage by restoring cell viability. The study also showed that AO-2 (1 µM) inhibited apoptosis and autophagy in cultured cortical neurons after exposure to OGD/R. This was demonstrated by a decrease in the percentage of dead cells as well as decreased expression of the autophagy-related protein (LC3-II) and cleaved caspase-3. These neuroprotective effects of AO-2 are mediated by activation of the AKT/mTOR signalling pathway [104]. Consistent with this, Liu et al. [105] also showed that pretreatment with the dextrorotatory enantiomer of alpinidinoid A [(+)-1] (0.5, 1, 5 µM) from *A. officinarum* for 4 h enhanced the cell viability of OGD/R-induced cortical neurons in vitro. The study also found that (+)-1 (5 µM) attenuated OGD/R-induced cell apoptosis in cultured cortical neurons via the PI3K/AKT/mTOR signalling pathway [105].

### 5.4. Effect of A. officinarum on Depression

Depression is a psychiatric disorder characterised by persistent low mood, lack of interest in activities, poor attention, altered appetite and sleep patterns, cognitive deficits, feelings of powerlessness, excessive guilt and suicidal thoughts [106,107]. The aetiology of pathological depression is complex, but dysregulation of monoaminergic neurotransmitters such as serotonin (5-HT), norepinephrine (NE) and dopamine (DA) has been identified as a key factor in the pathogenesis of depression. Several physiological processes, including motivation, emotion regulation, motor control, memory and learning, are mediated by monoaminergic neurotransmitters [107,108,109]. Most antidepressants are primarily designed to inhibit the reuptake and/or degradation of monoamines, thereby increasing the availability of monoamines in the synaptic space [110]. Hyperactivity of the hypothalamic–pituitary–adrenal axis (HPA axis) and inflammation also play a role in the aetiology of depression [111]. The HPA axis regulates the synthesis and secretion of the stress hormone cortisol. Dysregulation of the HPA axis leads to excessive production of cortisol, which subsequently restricts neurogenesis by reducing brain-derived neurotrophic factors (BDNF) in the hippocampus and impairing γ-aminobutyric acid (GABA)ergic and/or glutamatergic neurotransmission [109,112,113].

Numerous studies have shown that herbal remedies and their bioactive constituents have a positive effect in alleviating the symptoms of depression by modulating monoamine neurotransmitters, the HPA axis and neuroinflammation in human and in vivo models. The effect of *A. officinarum* extracts on in vivo models of depression has also been investigated. In this review, we discovered two in vivo studies that investigated the effect of *A. officinarum* on in vivo models of depression (Table 7). For example, Awari et al. investigated the antidepressant activity of an ethanol extract of *A. officinarum* (100, 200 and 400 mg/kg BW) by subjecting mice to the forced swim test (FST) and the tail suspension test (TST) [114]. The study showed that mice treated with 400 mg/kg BW *A. officinarum* for 15 days had a shorter immobility time in both the FST and the TST. The FST and TST are the most commonly used behavioural tests to assess depression-like behaviour in mice, and immobility in mice may indicate a depressive state similar to human depression [115,116]. The authors further confirmed that *A. officinarum* exerts its antidepressant effect by modulating the hypothalamic–pituitary–adrenal (HPA) axis, Na^+^, K^+^-ATPase, monoamines (NE, DA and 5-HT) and GABA in the brain. In addition, *A. officinarum* has shown similar antidepressant effects to the conventional drugs moclobemide and imipramine [114]. In another study, mice exposed to chronic unpredictable stimuli (CUS), a model for depression, were administered with hydroalcoholic extract of *A. officinarum* (50, 100 and 150 mg/kg BW) for 21 days. It was found that the administration of *A. officinarum* significantly reduced the duration of immobility in the FST and TST. In addition, treatment with *A. officinarum* extract resulted in a significant increase in antioxidant capacity and a significant decrease in MDA levels in the brain and serum, suggesting its contribution to antidepressant effects [117].

### 5.5. Effect of A. officinarum on Epilepsy and Seizure

Epilepsy is a chronic neurological disorder characterised by spontaneous and repetitive seizures caused by abnormal, hyperexcitable and hypersynchronous neuronal activity in the brain. This abnormal neuronal activity can be triggered by viral, structural or metabolic abnormalities [118,119,120]. There is evidence that oxidative stress, neuroinflammation and neurotransmitter dysfunction may play a role in the aetiology of epilepsy [120,121]. *A. officinarum* has been investigated for its potential in the treatment of epilepsy and seizures. Table 8 shows the effects of *A. officinarum* on epilepsy and seizures in two in vivo studies.

The GABAergic system has been identified as a potential target for the screening of anticonvulsant drugs. GABA is the primary inhibitory neurotransmitter in the brain and plays a major role in the modulation of neuronal excitability. Disruption of GABAergic neurotransmission is associated with various neurological disorders, including epilepsy [122]. *A. officinarum* has shown promise in modulating GABAergic neurotransmission. For example, Nejad et al. [123] has shown that pretreatment with the hydroalcoholic extract of *A. officinarum* (400 and 600 mg/kg BW) delays the onset of seizures and reduces the duration of seizures in mice induced with pentylenetetrazole (PTZ). Pretreatments with the flumazenil (a benzodiazepine antagonist) and naloxone (a nonselective opioid receptor antagonist) were able to block the effect of *A. officinarum* on seizures, suggesting that the GABAergic system and the opioid systems may have played a role in mediating these anticonvulsant effects of *A. officinarum* [123].

Patients with epilepsy often suffer from cognitive impairment [124] and depression [125]. Numerous evidence suggests that prolonged, repeated epileptic seizures can lead to the neuronal death in the hippocampus, entorhinal cortex, amygdala, thalamus and other limbic systems, contributing to cognitive and behavioural impairment [126,127,128]. Solati et al. investigated the effect of pretreatment with the hydroalcoholic extract of *A. officinarum* (50, 100 and 150 mg/kg) on the severity of epilepsy, memory impairment and depression in PTZ-induced seizures in rats [129]. Behavioural tests showed that pretreatment with *A. officinarum* reduced the severity of epileptic seizures, improved memory function and reduced depression. Similar to the study by Nejad et al. [123], treatment with flumazenil significantly reduced the anticonvulsant effect of *A. officinarum* extract (150 mg/kg), suggesting that the anticonvulsant effect of *A. officinarum* extract, along with its antiamnesic and antidepressant properties, may be mediated by modulation of the GABAergic system [129].

Oxidative stress plays a role in the pathogenesis of epileptic seizures. A number of experimental studies have shown that antiepileptic agents with antioxidant properties promote neuroprotection and attenuate epilepsy in animal models [121,130]. Interestingly, the studies by Nejad et al. [123] and Solati et al. [129] suggest that the potential anticonvulsant effect of the *A. officinarum* may be due to its antioxidant property. Nejad et al. suggested that the potential anticonvulsant effect of *A. officinarum* is related to the presence of apigenin and curcumin, which are known for their antioxidant effects [123]. Solati et al. reported that treatment with the hydroalcoholic extract of *A. officinarum* decreased brain and blood MDA levels while increasing brain antioxidant capacity in PTZ-induced seizures in rats [129].

**Table 8:** Summary of the effects of *A. officinarum* on epilepsy in in vivo studies.

Study Design	Plant Extract/Bioactive Compound	Treatment Dosage	Duration of Study	Findings	References
In vivo studies					
Male albino mice induced with PTZ (60 mg/kg, i.p.)	*A. officinarum* extract (hydroalcoholic)	200, 400 and 600 mg/kg, i.p. (30 min before PTZ induction)	-	↑ the onset of seizure. ↓ the duration of seizure. Flumazenil and naloxone pretreatments inhibited the anticonvulsant activity of *A. officinarum*.	[123]
Male Wistar rats induced with PTZ (35 mg/kg i.p.) daily every 48 h for 9 days and PTZ (60 mg/kg i.p.) on the 10th day.	*A. officinarum* extract (hydroalcoholic)	50, 100 and 150 mg/kg, i.p. (30 min before PTZ induction)	10 days	↑ survival rate. ↑ onset of seizure. ↓ duration of seizure. ↓ total frequency of entire body seizures. ↓ total frequency of repeated spinning and jumping. ↓ immobility time in TST. Improved passive avoidance memory test. ↑ serum antioxidant capacity. ↓ serum and brain MDA level. Flumazenil pretreatment inhibited the anticonvulsant activity of *A. officinarum*.	[129]

Upward arrow (↑) indicate increased; Downward arrow (↓) indicate decreased.

### 5.6. Effect of A. officinarum on Nociceptive Pain

Rheumatoid arthritis (RA) is a chronic systemic autoimmune disease characterised by inflammation and pain, mainly affecting the joints and surrounding tissues [131]. Nociceptive pain is pain caused by the stimulation of nociceptors (pain receptors) in non-neural tissues such as skin, joints and muscles due to inflammation or injury [132,133,134]. Signals from the nociceptors are transmitted from the peripheral neurons via the spinothalamic tract to the dorsal root ganglion (DRG) of the spinal cord and finally to the brain, resulting in pain perception [135]. The primary treatment plan for rheumatoid arthritis is to suppress inflammation, relieve pain and prevent joint destruction [136].

The antinociceptive effect of *A. officinarum* in an in vivo model of chronic arthritis was also investigated (Table 9). Lee et al. investigated the antinociceptive effect of an 80% ethanolic extract of *A. officinarum* in a model of chronic arthritis induced by a complete Freund’s adjuvant (CFA) [137]. The study showed that a 21-day treatment with *A. officinarum* (200 and 500 mg/kg BW) attenuated CFA-induced inflammatory pain-like behaviour in rats, as evidenced by a significant improvement in paw withdrawal latency (PWL) in response to thermal stimuli and a reduction in ankle flexion scores. The study also found that treatment with *A. officinarum* restored c-Fos expression in the hippocampus of CFA-induced rats, with the highest expression in the dentate gyrus region, suggesting that *A. officinarum* has a psychiatric effect [137]. The hippocampus plays a role in pain processing and the modulation of nociceptive stimuli [138]. Previous studies have reported that chronic pain impairs synaptic plasticity of the hippocampal mossy fibre CA3 and neurogenesis of the dentate gyrus. These pathophysiological changes can lead to cognitive deficits as well as anxiety- and depression-like behaviours [139,140,141].

## 6. Conclusions and Future Direction

Taken together, this review provided important insights into the therapeutic potential of *A. officinarum* and its bioactive compounds in the treatment of neurological disorders. Several studies on *A. officinarum* and its bioactive compounds show its pharmacological activities in a number of neurological disorders. Despite the widespread use of *A. officinarum* extract and its bioactive compounds in various models of neurological disorders, the actual mechanisms underlying the therapeutic effects are known to be diverse but poorly understood. Some studies have found considerable therapeutic effects in in vitro studies, but the evidence from in vitro studies needs to be confirmed in in vivo studies. For example, in vitro studies suggest that *A. officinarum* extract and its diarylheptanoids can treat and prevent AD through various mechanisms such as antioxidation, anti-apoptosis, anti-amyloid and anticholinesterase. However, further studies on brain tissue and cognitive function in animal models of AD should be conducted to confirm the claims described. Although *A. officinarum* extract has been reported to improve memory impairments in the PTZ seizure rat model, further studies on the effects of *A. officinarum* on cognition should be conducted using different protocols involving all forms of memory, such as the Morris water maze, open-field test, T-maze and radial arm maze, to identify the specific cognitive areas that respond best to *A. officinarum*. To validate the effect of *A. officinarum* on memory restoration, further experiments are needed to measure the levels of neurotransmitters (such as acetylcholine and dopamine) and neurotrophic factors (such as nerve growth factor-NGF and brain-derived neurotrophic factor) involved in learning and memory functions and to assess long-term potentiation (LTP) in the hippocampal regions. In addition, further experiments should be conducted to prove that *A. officinarum* is a potential herbal medicine for AD by evaluating its effect in attenuating the accumulation of Aβ and phosphorylated tau protein in AD brain tissue.

**Figure 4:**
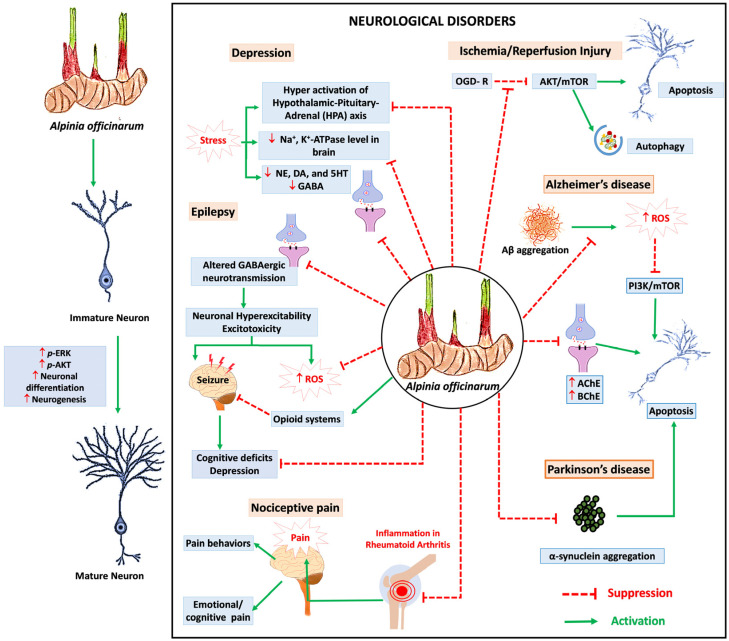
Proposed therapeutic effects of *Alpinia officinarum* in central nervous system (CNS).

*A. officinarum* has also been shown to attenuate the biological hallmark of PD by inhibiting α-synuclein aggregation. However, future studies are needed to evaluate the motor functions, neurochemical changes and pathological changes associated with PD in an in vivo model. Despite the promising neuroprotective properties of *A. officinarum* reported in previous studies, it is unclear how *A. officinarum* reaches and specifically acts on neural cells. Therefore, it is important to investigate the ability of *A. officinarum* to cross the BBB and its affinity for CNS receptors to gain a better understanding of its efficacy as a therapeutic agent in CNS disorders. There is also a lack of human clinical trials to confirm the safety and efficacy of *A. officinarum* in neurological disorders. Future studies need to include a comprehensive assessment of adverse effects, investigation of the effects of long-term use and evaluation of possible interactions with other medicinal products. The proposed therapeutic effects of *A. officinarum* in the CNS are shown in Figure 4.

## Figures and Tables

**Table 1:** Summary of the phytochemical compounds isolated from *A. officinarum*.

Type	Compound	Reference
Phenylpropanoid	(4E)-1,5-bis(4-hydroxyphenyl)-1-methoxy-2-(methoxymethyl)-4-pentene; (4E)-1,5bis(4-hydroxyphenyl)-2-(methoxymethyl)-4-penten-1-ol; (4E)-1,5-bis(4-hydroxyphenyl)-1-ethoxy-2-(methoxymethyl)-4-pentene; (4E)-1,5bis(4-hydroxyphenyl)-2-(hydroxymethyl)-4-penten-1-ol; (4E)-1,5-bis(4-hydroxyphenyl)-1-[(2E)-3-(4-acetoxyphenyl)-2-propenoxy]-2-(methoxymethyl)-4-pentene; (E)-p-coumaryl alcohol-O-methylether; trans-p-Coumaryl alcohol	[43]
Flavanoids	Flavonols	
	Galangin; galangin-3-methylether; kaempferol; kaempferide; quercetin	[46]
	Apigenin	[47]
	Flavanonols	
	Pinocembrin; pinobaksin; epicatechin	[46]
Diarylheptanoid	Linear	
	(4*E*)-1,7-diphenyl-4-en-3-heptanone; 7-(4-hydroxylphenyl)-1-phenyl-4-en-3-heptanone; 7-(4-hydroxyl-3-methoxyphenyl)-1-phenyl-4-en-3-heptanone or 7-(4-Hydroxy-3-methoxyphenyl)-1-phenyl-4E-hepten-3-one	[48]
	(4*E*)-1,7-diphenylhept-4-en-3-one; (4*E*)-7-(4-hydroxy-3-methoxyphenyl)-1-phenylhept-4-en-3-one;5-hydroxy-1,7-diphenylheptan-3-one; 5-hydroxy-7-(4-hydroxy-3-methoxyphenyl)-1-phenylheptan-3-one	[49]
	(4*E*)-7-(3,4-dihydroxylphenyl)-1-(4-hydroxyl-3-methoxyphenyl)-4-en-3-heptanone; (5*R*)-1-(3,4-dihydroxyphenyl)-5-hydroxy-7-(4-hydroxy-3-methoxy-phenyl)-3-heptanone; (5*S*)-7-(3,4-dihydroxyphenyl)-5-hydroxy-1-phenyl-3-heptanone	[50]
	(*E*)-7-(4-hydroxy-3-methoxyphenyl)-1-(hydroxyphenyl) hept-4-en-3-one; (*R*)-5-hydroxy-7-(4-hydroxy-3-methoxyphenyl)-1-phenyl-3-heptanone; (*S*)-5-hydroxy-7-(4-hydroxyphenyl)-1-phenylheptan-3-one; (5*S*)-1-(4-hydroxyphenyl)-5-hydroxy-7-(4-hydroxy-3 -methoxy-phenyl)-3-heptanone; (5*S*)-1,7-diphenyl-5-methoxy-3-heptanone; (*S*)-7-(4-hydroxyphenyl)-5-methoxy-1-phenylheptan-3-one; (*R*)-7-(4-hydroxy-3-methoxy phenyl)-5-methoxy-1-phenylheptan-3-one; (*S*)-7-(4-hydroxy-3-methoxyphenyl)-1-(4-hydroxyphenyl)-5-methoxyheptan-3-one; (3*R*,5*R*)-1-(4-hydroxy-3-methoxyphenyl)-7-phenyl-3,5-heptanediol; (*S*,*E*)-2-hydroxy-1,7-diphenylhept-4-en-3-one	[51]
	(4*E*, 6*R*)-6-hydroxy-7-(4-hydroxy-3-methoxyphenyl)-1-phenyl-4-en-3-heptanone; (4*E*, 6*R*)-6-hydroxy-1,7-diphenyl-4-en-3-heptanone	[52]
	(5*R*)-1-(4-hydroxy-3-methoxy-phenyl)-5-hydroxy-7-(4-hydroxy phenyl)-3-heptanone; (5*R*)-1-(4-hydroxyphenyl)-5-hydroxy-7-(4-hydroxy-3-methoxy-phenyl)-3-heptanone; 1-(4-hydroxy-3-methoxyphenyl)-7-phenyl-3,5-heptanediol; (3*S*,5*S*)-1-(4-hydroxyphenyl)-7-phenyl-3,5-heptanediol	[53]
	5(*S*)-acetoxy-7-(4-dihydroxyphenyl)-1-phenyl-3-heptanone rhizomes	[54]
	(4*Z*,6*E*)-5-hydroxy-1-(4-hydroxy-3-methoxyphenyl)-7-phenylhepta-4,6-dien-3-one	[46]
	(5*R*)-5-hydroxy-1,7-diphenylheptan-3-one; (5*R*)-7-(4-hydroxy-3-methoxyphenyl)-5-methoxy-1-phenylheptan-3-one; (5R)-5-ethoxy-7-(4-hydroxy-3-methoxyphenyl)-1-phenylheptan-3-one	[51,55]
	(4*E*)-7-(4-hydroxyphenyl)-1-phenyl-4-hepten-3-one; (4*E*)-7-(4-hydroxy-3-methoxyphenyl)-1-phenyl-hept-4-en-3-one; (5*R*)-7-(4-hydroxy-3-methoxyphenyl)-5-methoxy-1-phenyl-3-heptanone	[56]
	Cyclic	
	Alpinoid A, B, C, D; 3,6-furan-1,7-diphenylheptane	[51]
	Dimeric-diarylheptanoids	
	Alpinin A, B, C, D	[57,58]
	Novel	
	Officinaruminane B	[54]
Glycosides	(*1R*,3*S*, 4*S*)-trans-3-hydroxy-1,8-cineole β-d-glucopyranoside; 3-methyl-but-2-en-1-yl β-d-glucopyranoside; benzyl β-d-glucopyranoside, chavicol β-glycoside; chavicol β-rutinoside; 1-hydroxy-2-O-β-d-glucopyranosyl-4-allylbenzene; demethyleugenol β-dglucopyranoside; demethyleugenol β-rutinoside; chavicol β-rutinoside; 1,2-di-O-β-d-glucopyranosyl-4-allylbenzene	[43]
	alpinoside A; *n*-butyl-β-d-fructofuranoside	[46]
Sesquiterpenes and Diterpenes	Alpiniaterpene A	[59]
	(Z)-12, 14-labdadien-15(16)-olide-17-oic acid; 4-isopropyl-6-methyl-1-naphthalenemethanol	[60]

**Table 2:** Phytochemical compounds and biological activities of the different parts of *A. officinarum* plant.

Plant Part	Extraction/Isolation Method	Major Phytochemical Compounds	Potential Biological Activity	References
Aerial parts	Not mentioned	Galangin, 3-O-Methylgalangin, Pinocembrin, Pinobanksin, Kaempferide	Not mentioned.	[61]
Aerial parts and rhizome	Maceration (methanol) and ultrasonic extraction	Nootkatone, Diarylheptanoids, Chrysin, Pinocembrin, Tectochrysin, Apigenin, Galangin, Acacetin, Quercetin, Isorhamnetin, Hannokinol, Izalpinin, Rutin, Yakuchinone A, Hexahydrocurcumin, Luteolin, Kaempferol, Kaempferide	Not mentioned.	[62]
Rhizome	Maceration (methanol) and solvent partition	1,7-diphenylhept-4-en-3-one, 5-hydroxy-1,7-diphenyl-3-heptanone, Galangin, Kaempferide, 5-hydroxy-7-(4″-hydroxy-3″-methoxyphenyl)-1-phenyl-3-heptanone	Galangin and 5-hydroxy-7-(4″-hydroxy-3″-methoxyphenyl)-1-phenyl-3-heptanone exhibit anti-inflammatory and antioxidant activities.	[63]
Rhizome	Solvent partition (80% acetone extract)	5-hydroxy-1,7-diphenyl-3-heptanone, 7-(4″-hydroxy-3″-methoxyphenyl)-1-phenylhept-4-en-3-one, 5-hydroxy-7-(4″-hydroxy-3″-methoxyphenyl)-1-phenyl-3-heptanone, 3,5-dihydroxy-1,7-diphenylheptane, Kaempferide, Galangin	Anti-proliferative inhibition of nitric oxide production enzyme and transcription factor inhibitor.	[64,65]
Rhizome	Solvent partition (ethanol extract)	Alpinin B, 1,7-diphenyl-3,5-heptanedione, (4E)-1,7-diphenylhept-4-en-3-one, (4E)-7-(4-hydroxyphenyl)-1-phenylhept-4-en-3-one, Alpinin C, Alpinin D	Anticancer	[57,66]
Taproot, aerial and fibril	Maceration (methanol) and ultrasonic extraction	5R-hydroxy-7-(4-hydroxy3-methpxyphenyl)-1-phenyl-3-heptanone), Kaempferide, Galangin	Antioxidant	[67]

**Table 3:** Summary of the effects of *A. officinarum* on neuronal cells in in vitro and in vivo studies.

Study Design	Plant Extract/Bioactive Compound	Treatment Dosage	Duration of Study	Findings	References
In vitro studies					
Human neuroblastoma cells, NB-39 cells (undifferentiated neurons).	Diarylheptanoids: 7-(4-hydroxy-3-methoxyphenyl)-1-phenyl-4E-hepten-3-one (5R)-5-methoxy-7-(4-hydroxy-3-methoxyphenyl)-1-phenyl-3-heptanone	10^−8^ M	48 h	↑ neurite outgrowth and branching in neurons.	[68]
Mouse neuroblastoma cells, Neuro-2a cells and primary hippocampal cells.	Diarylheptanoids: 7-(4-hydroxy-3-methoxyphenyl)-1-phenyl-4E-hepten-3-one 7-(4-hydroxy-3-methoxyphenyl)-1-phenyl-4E-hepten-3-one (5S)-5-hydroxy7-(3,4-dihydroxyphenyl)-1-phenyl-3-heptanone	2–4 μM	24 h	↑ neurite length in Neuro-2a and hippocampal cells. ↑ differentiation rate in Neuro-2a. ↑ percentage of axon-bearing cells in hippocampal cells. ↑ expression of neurofilament-M. ↑ phosphorylation of Thr308 and Ser473 residues. No effect on JNK signalling pathway.	[55]
In vivo study					
Healthy male C57BL/6 mice.	Diarylheptanoids: 7-(4-hydroxy-3-methoxyphenyl)-1-phenyl-4E-hepten-3-one 7-(4-hydroxy-3-methoxyphenyl)-1-phenyl-4E-hepten-3-one (5S)-5-hydroxy7-(3,4-dihydroxyphenyl)-1-phenyl-3-heptanone	28 mg/kg/daily i.p.	14 days	No effect on the proliferation of progenitor cells. ↑ neuN-positive cells in the adult dentate gyrus. ↓ DCX-positive cells in the adult dentate gyrus. ↑ *p*-ERK and *p*-Akt levels.	[55]

Upward arrow (↑) indicate increased; Downward arrow (↓) indicate decreased.

**Table 4:** Summary of the effects of *A. officinarum* on Alzheimer’s disease in in vitro studies.

Study Design	Plant Extract/Bioactive Compound	Treatment Dosage	Duration of Study	Findings	References
In vitro studies					
Anticholinergic assay	*A. officinarum* extract (Aqueous, ethanol, Aqueous-ethanol extracts)	-	-	↑ AChE inhibition activity. ↑ Fe^3+^ and Cu^2+^ reducing capability. ↑ DPPH radical scavenging activity.	[78]
Primary hippocampal cells induced with Aβ_42_ peptides (1 µM) for 24 h.	Diarylheptanoids: 7-(4-hydroxyphenyl)-1-phenyl-4E-hepten-3-one (AO-1)	0.5 µM	1 h	↓ dendritic impairment. ↓ number of apoptotic cells. ↓ caspase-3 activity. ↓ ROS level. Activates PI3K-mTOR signalling pathway. No effect on MEK activity.	[75]
PC12 cells and primary hippocampal cells induced with Aβ_42_ peptides (1 µM) for 24 h.	Diarylheptanoids: 7-(4-Hydroxy-3-methoxyphenyl)-1-phenyl-4E-hepten-3-one (AO-2)	0.5–4 µsM	2 h	↑ PC12 cell viability. ↓ apoptosis and necrosis in PC12 and hippocampal cells. ↓ caspase-3 activity. ↓ ROS level. ↓ dendritic impairment. Activates PI3K-mTOR signalling pathway.	[76]
AChE and BChE assays.	Diarylheptanoids: (-)-alpininoid B, (4E)-1,7-diphenyl-4-hepten-3-one (4E)-7-(4-hydroxyphenyl)-1-phenyl-4-hepten-3-one (4E)-7-(4-hydroxy-3-methoxyphenyl)-1-phenyl-hept-4-en-3-one Dihydroyashsbushiketol (5R)-7-(4-hydroxy-3-methoxyphenyl)-5-methoxy-1-phenyl-3-heptanone Flavonoids: Kaempferide Galangin	-	-	↑ AChE inhibition activity. Weak to none BChE activity.	[56]
Human neuroblastoma (Sh-SY5Y) cells induced with H_2_O_2_ for 24 h.	Several diarylheptanoid compounds from *A. officinarum* (compounds **7**, **10**, **12**, **20**, **22**, **25**, **28**, **33**, **35**, **37** and **42**)	5, 10 or 20 μM		↑ cell viability. ↓ MDA and NO production. ↓ ROS level.	[79]

Upward arrow (↑) indicate increased; Downward arrow (↓) indicate decreased.

**Table 6:** Summary of the effects of *A. officinarum* on ischaemia-reperfusion in in vitro studies.

Study Design	Plant Extract/Bioactive Compound	Treatment Dosage	Duration of Study	Findings	References
In vitro studies					
Primary cortical neurons exposed to 4 h of oxygen–glucose deprivation and 24 h of reoxygenation (OGD/R model).	Diarylheptanoids: 7-(4-Hydroxy-3-methoxyphenyl)-1-phenyl-4E-hepten-3-one (AO-2)	0.25, 0.5 and 1 µM	2 h	↑ cell viability. ↓ cell apoptosis. ↓ LC3-II and cleaved saspase-3 activities. ↑ activation of AKT/mTOR signalling pathway.	[104]
Primary cortical neurons exposed to 4 h of oxygen–glucose deprivation and 24 h of reoxygenation (OGD/R model).	Diarylheptanoids: Alpinidinoids A [(+)-1]	0.5, 1 and 5 µM	4 h	↑ cell viability. ↓ cell apoptosis. ↓ cleaved caspase-3 activity. ↑ activation of PI3K/AKT/mTOR signalling pathway.	[105]

Phosphatidylinositol 3-kinase (PI3K)-mammalian target of rapamycin (mTOR) pathways. Upward arrow (↑) indicate increased; Downward arrow (↓) indicate decreased.

**Table 7:** Summary of the effects of *A. officinarum* on depression in in vivo studies.

Study Design	Plant Extract/Bioactive Compound	Treatment Dosage	Duration of Study	Findings	References
In vivo studies					
Male Swiss albino mice subjected to tail suspension (TST) and forced swimming (FST) tests to induce depression-like behavior.	*A. officinarum* extract (methanol)	100, 200 and 400 mg/kg/day p.o.	15 days	↓ immobility time in TST and FST. ↑ Na^+^, K^+^-ATPase level in brain in TST. ↓ plasma corticosterone level. ↑ monoamine (NE, DA and 5HT) level in brain in FST. ↑ gamma-amino-butyric acid (GABA) level in brain in FST.	[114]
Male BALB/c mice exposed to chronic unpredictable stressors (CUS) for 3 weeks.	*A. officinarum* extract (hydroalcoholic)	50, 100 and 150 mg/kg/day i.p.	21 days	↓ immobility time in TST and FST. No effect on rotarod test. ↓ MDA level in brain and serum. ↑ FRAP level in brain and serum.	[117]

Norepinephrine (NE); dopamine (DA); 5-hydroxytryptamine (5HT); malondialdehyde (MDA); ferric reducing antioxidant power (FRAP). Upward arrow (↑) indicate increased; Downward arrow (↓) indicate decreased.

**Table 9:** Summary of the effects of *A. officinarum* on nociceptive pain in an in vivo study.

Study Design	Plant Extract/Bioactive Compound	Treatment Dosage	Duration of Study	Findings	References
In vivo study					
Male Sprague–Dawley (SD) rats induced with complete Freund’s adjuvant with Mycobacterium butyricum 1% suspension in mineral oil (S.C.)	*A. officinarum* extract (ethanol)	200 and 500 mg/kg day p.o.	21 days	↑ paw withdrawal latency (PWL). ↓ ankles flexion scores. ↑ c-Fos in brain hippocampus. Dentate gyrus showed highest c-Fos expression compared to C1, C2 and C3 regions.	[137]

Upward arrow (↑) indicate increased; Downward arrow (↓) indicate decreased.
